# Anticoagulation-Related Dilemma: A Thrombocytopenic Patient With Numerous Thrombogenic Risk Factors

**DOI:** 10.7759/cureus.98467

**Published:** 2025-12-04

**Authors:** Timothy Johnson, Jennifer Trube, Juan O Rodriguez-Padilla, Mark Soliman, Parker Williams, Noah Kosnik, Christian Abreu-Ramirez

**Affiliations:** 1 Internal Medicine, Lakeland Regional Health, Lakeland, USA; 2 Anesthesiology, Lakeland Regional Health, Lakeland, USA; 3 Pulmonary Critical Care, Lakeland Regional Health, Lakeland, USA

**Keywords:** anticoagulation, atrial fibrillation, cancer, immunotherapy, pancytopenia, prothrombotic risk, risk factors, st-elevation myocardial infarction (stemi), thrombocytopenia, transfusion

## Abstract

Anticoagulation is typically withheld in cases of severe thrombocytopenia, but clinical decision-making becomes challenging when a patient has multiple established venous and arterial thrombogenic risk factors, along with a fluctuating platelet level. Given the absence of clear guidelines and with an uncertain balance of risks and benefits, this case report and the accompanying discussion may offer useful insights for managing similar situations in the future.

A 70‑year‑old male with metastatic adenocarcinoma of the lung presented to the emergency department for evaluation of worsening fatigue and weakness, which had led to a presyncopal episode with a ground-level fall. His cancer had been diagnosed one year prior and treated monthly with radiation, chemo, and immunotherapies. On admission, he was hypotensive, tachycardic, febrile, and markedly pancytopenic. CT imaging suggested pneumonia superimposed on an extensive tumor burden. He was also found to have new-onset atrial fibrillation (AF) with a rapid ventricular rate. This was initially rate-controlled with labetalol, but anticoagulation was held due to thrombocytopenia, despite a CHADS₂/VASc score of 2. He was transferred to the ICU for chronic obstructive pulmonary disease exacerbation and septic shock complicated by pancytopenia and neutropenic fever.

The patient was treated with broad‑spectrum antibiotics, steroids, filgrastim, vasopressors, intubation, and multiple transfusions (three units of platelets and one unit of packed red blood cells) during the first three days of his five-day admission. Platelet counts ranged between 11 and 61 × 10³/µL despite interventions. On day three, blood cultures grew Pseudomonas; antibiotics were narrowed, and he was successfully extubated to bilevel positive airway pressure. By day four, his atrial fibrillation converted to sinus rhythm for the first time during this admission, and digoxin was initiated due to ongoing hypotension. Subsequently, he developed a complicated pleural effusion that required chest tube placement. Unfortunately, on day five of admission, he exhibited ST‑segment elevations (V2-V6) consistent with anterior myocardial infarction. Due to thrombocytopenia, comorbidities, and goals of care, anticoagulation and reperfusion interventions were deferred. Care transitioned to comfort measures only, and he died 48 hours later.

This report highlights a unique instance of a common clinical dilemma: determining whether to initiate or withhold anticoagulation in a thrombocytopenic patient with multiple high-risk prothrombotic factors. To our knowledge, no established guidelines specifically address the complex overlap of severe, fluctuating thrombocytopenia and elevated thrombotic risk. This report also underscores the importance of individualized clinical judgment and continual reassessment when making anticoagulation decisions.

## Introduction

Anticoagulation is typically withheld in cases of severe thrombocytopenia, yet decision-making becomes difficult when a patient also has multiple established venous and arterial thrombogenic risk factors, along with a fluctuating platelet level. Thrombocytopenia plays a significant role in outcomes among ICU patients, with affected patients exhibiting a 15% higher mortality rate than those with normal platelet counts, and an even greater risk when platelet levels drop by ≥30% [1]. However, treating ICU patients without anticoagulation carries its own risks, with the incidence of venous thromboembolism reported between 15-60% of patients [2]. Several large cohort studies and meta-analyses have shown that omission of early anticoagulation (within 24 hours of ICU admission) raises hospital mortality by roughly 4-15%, depending on underlying comorbidities [3].

Published guidelines often recommend withholding anticoagulation when platelet counts fall below 25 × 10³/µL, recommending individualized risk assessment and consideration of half-dose anticoagulation between 25-50 × 10³/µL, with full anticoagulation typically resumed once counts rise above 50 × 10³/µL [4,5]. The middle zone, where the patient in this case report often fell, relies heavily on provider judgment to use risk/benefit analysis in terms of providing anticoagulation. In the absence of definitive guidance and with an uncertain balance between thrombotic and bleeding risk, this case report and the accompanying discussion may offer valuable insight for future similar scenarios.

## Case presentation

**Figure 1:**
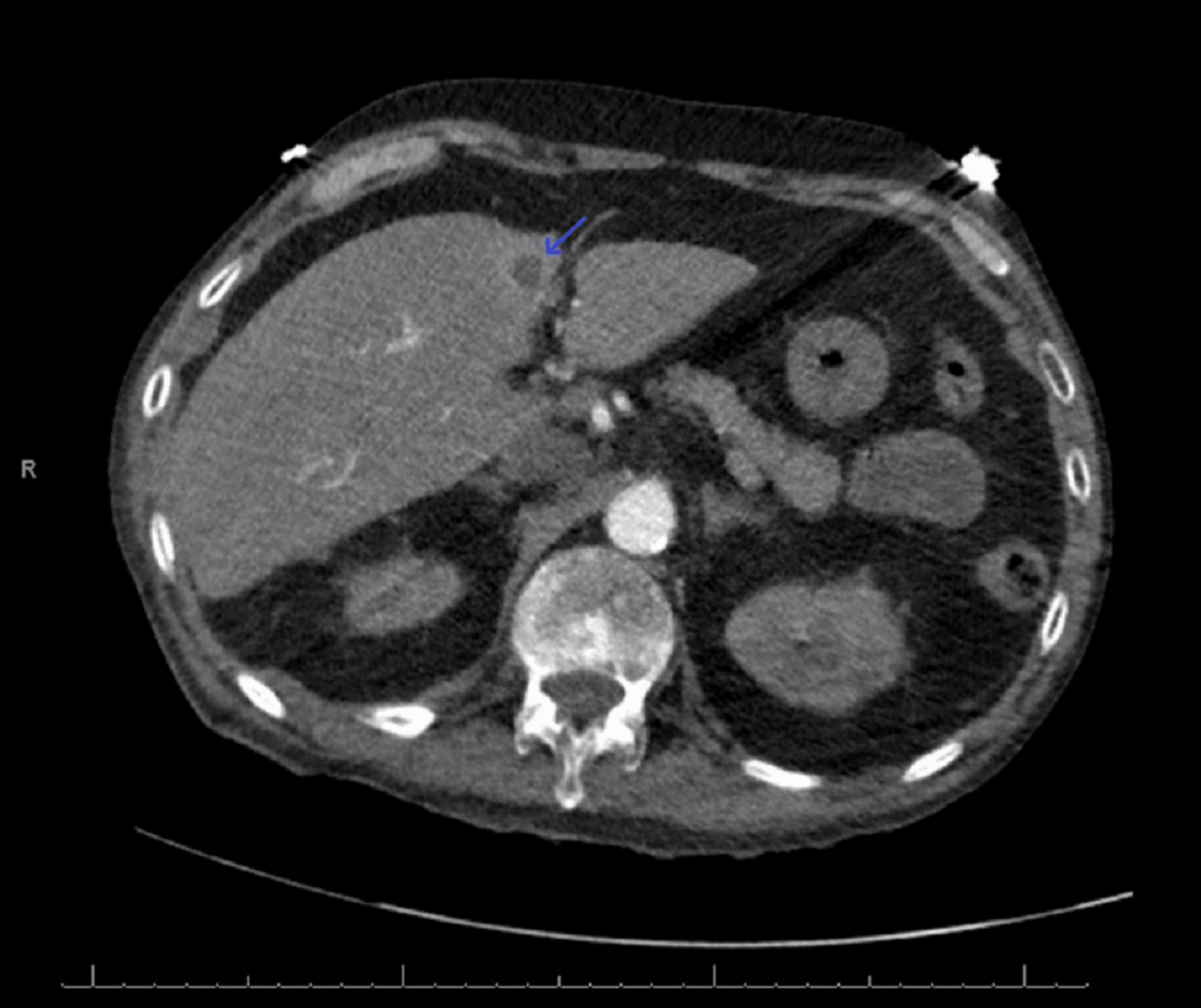
CTA chest with contrast Arrow: metastasis in the liver CTA: computed tomography angiography

A 70-year-old male with a history of essential hypertension, hyperlipidemia, resting tremor, benign prostatic hypertrophy, a 100+ pack-year smoking history, prior alcohol abuse, and metastatic lung adenocarcinoma presented to the emergency department with worsening fatigue and weakness, culminating in a presyncopal episode and ground-level fall. His cancer had metastasized to the supraclavicular and right hilar lymph nodes and to the liver (Figure 1). He had been diagnosed one year prior and treated monthly with radiation, chemo, and immunotherapies.

**Figure 2:**
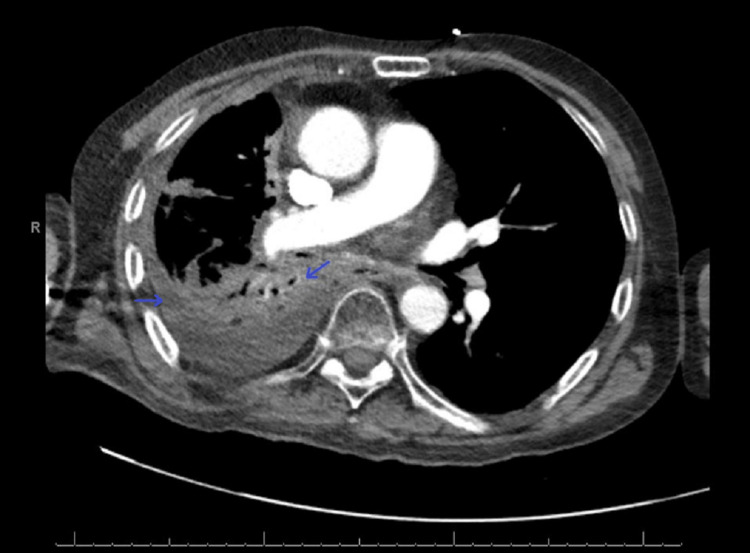
CTA chest with contrast Arrow: pneumonia superimposed on an extensive tumor burden CTA: computed tomography angiography

**Figure 3:**
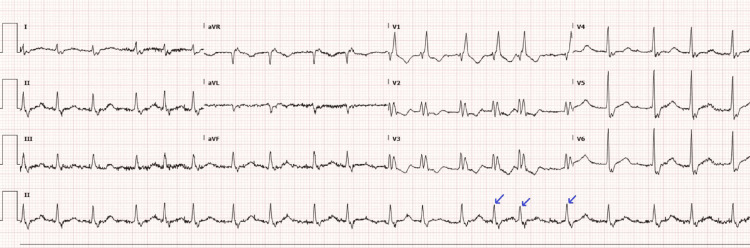
EKG on admission Arrow: atrial fibrillation with rapid ventricular response EKG: electrocardiogram

On admission, the patient was hypotensive, tachycardic, febrile, and markedly pancytopenic. CT imaging suggested pneumonia superimposed on an extensive tumor burden (Figure 2). EKG revealed new-onset atrial fibrillation (AF) with rapid ventricular response and right bundle branch block (Figure 3). This was initially rate-controlled with metoprolol, but without anticoagulation (CHADS₂/VASc = 2), due to thrombocytopenia (HAS-BLED = 5 (high risk); Khorana Score = 2 (intermediate risk)). Despite significant fluid resuscitation, he remained persistently hypotensive, requiring vasopressors and admission to the ICU. He was admitted for chronic obstructive pulmonary disease exacerbation and septic shock complicated by pancytopenia and neutropenic fever.

**Figure 4:**
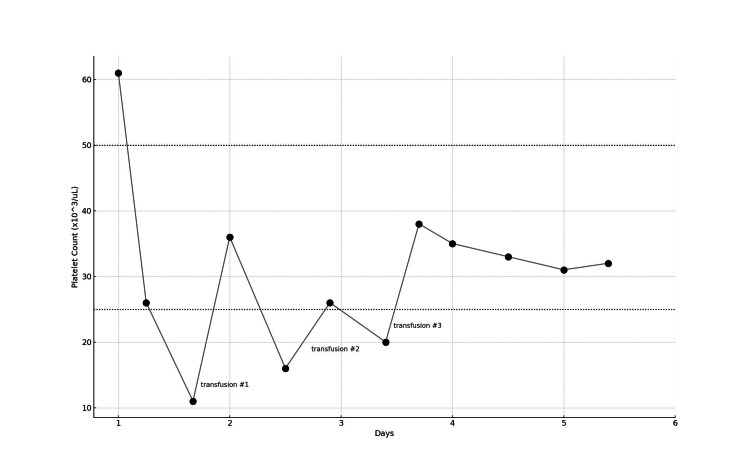
Platelet fluctuation and transfusions The figure demonstrates a timeline of recorded platelet levels and required platelet transfusions during the patient's five-day intensive care unit stay

During the first two days of his ICU stay, he was started on vancomycin, meropenem, econazole, methylprednisolone, scheduled breathing treatments, and filgrastim. He developed increased work of breathing. The subsequent bilevel positive airway pressure (BiPAP) trial was unsuccessful, necessitating intubation. Additionally, multiple transfusions (three units of platelets and one unit of packed red blood cells) were required during the first three days of his five-day admission. Platelet counts ranged from 11 to 61 × 10³/µL despite interventions (Figure 4).

On ICU Day three, blood cultures grew Pseudomonas, and antibiotics were narrowed to cefepime. He was also successfully extubated to BiPAP. On ICU day four, a complicated pleural effusion developed, requiring chest tube placement. Due to persistent hypotension, rate control with metoprolol was discontinued. Instead, rhythm control with digoxin was initiated. His AF converted to sinus rhythm for the first time during this admission.

**Figure 5:**
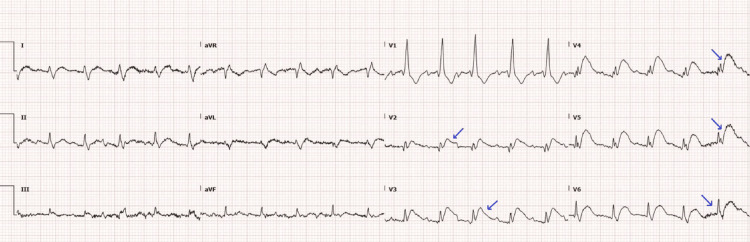
EKG on ICU day five Arrows: ST-segment elevations in V2–V6 EKG: electrocardiogram; ICU: intensive care unit

On ICU day five, the patient developed sudden-onset chest pain, anxiety, and disorientation. Subsequent EKG exhibited ST‑segment elevations (V2-V6) consistent with anterior myocardial infarction (Figure 5). Cardiology was consulted. However, due to his thrombocytopenia, comorbidities, and goals of care, anticoagulation and reperfusion interventions were deferred. His care was transitioned to comfort measures only, and he was transferred to the palliative care unit. He died 48 hours later.

## Discussion

This report highlights a unique instance of a common clinical dilemma: determining whether to initiate or withhold anticoagulation in a thrombocytopenic patient with multiple high-risk prothrombotic factors. This patient’s platelet level fluctuated significantly, creating ambiguity. Additionally, his comorbidities included many documented risk enhancers for both arterial and venous thromboembolism.

Rationale for withholding anticoagulation

As stated, the patient’s platelet levels fluctuated across all three previously noted thresholds. Although the patient had thrombocytopenia at baseline, likely due to chemotherapy (~60 × 10³/µL in the previous year), his levels during admission were even lower. He required three platelet transfusions during his ICU course, the last occurring on ICU day three. There was no evidence of bleeding or disseminated intravascular coagulation. Although the platelet level fluctuated, it was most often in the middle category, indicating individualized, clinical judgement for anticoagulation [4,5].

Rationale for anticoagulation

Active metastatic adenocarcinoma, prolonged immobility, recent invasive procedure, new-onset AF broken after >48 hours of sustained arrhythmia, and recent chemotherapeutic and immunotherapeutic treatments are all well-documented risk enhancers for both arterial and venous thromboembolism. The diagnosis of adenocarcinoma of the lung with metastasis has been shown to significantly increase the risk of thrombus formation. One prospective study demonstrated “pronounced blood hypercoagulability in ambulatory patients with lung adenocarcinoma, characterized by decreased Procoag‐PPL clotting time, enhanced endothelial cell activation", stimulating increased fibrinolytic activity and impaired thrombin generation [6,7].

It is well established in the literature that immobilized patients, especially in the geriatric population, are at increased risk of thrombus formation [8]. This further increased this patient’s risk. Although less studied, recent invasive procedures (i.e., chest tube) can increase the risk of thrombus through indirect mechanisms, thereby increasing hospital stay/immobility, local trauma, and inflammation. He had a new-onset AF with a CHADS₂/VASc score of 2, indicating long-term anticoagulation. Additionally, during this hospitalization, AF was sustained for >48 hours and subsequently returned to sinus rhythm, increasing the risk of ejecting a formed clot [9,10].

Finally, this patient had been receiving chemotherapy and immunotherapy monthly for over one year. This included several rounds of pemetrexed, pembrolizumab, carboplatin, paclitaxel, etoposide, and atezolizumab, many of which confer an increased thrombotic risk to the patient. Immune checkpoint inhibitors (pembrolizumab and atezolizumab) have been associated with a significantly increased risk for arterial thromboembolism [11]. Carboplatin is also shown to have a high incidence of thromboembolic events and arterial thrombosis, especially within four weeks of its last dose [12]. Pemetrexed has similarly been shown to increase the incidence of thromboembolic events [13].

Deliberation

Although we cannot confirm causality without troponin testing, cardiac catheterization, or an autopsy, the myocardial infarction may have been thrombus-mediated. After the patient transitioned to comfort measures-only care, several factors could have contributed to his death. The exact cause of myocardial infarction and death was never officially established.

To our knowledge, no established guideline addresses this complex intersection of significant, fluctuating thrombocytopenia and high thrombotic risk. In retrospect, we would advocate starting anticoagulation, at least at half dose, for patients with non-transfusion dependent thrombocytopenia >25 × 10³/µL with several well-studied, active thrombogenic risk factors.

## Conclusions

This report highlights the critical need for individualized clinical judgment and constant reassessment relating to anticoagulation. Although the precise cause of this patient’s unfortunate death remains uncertain, the clinical dilemma remains highly relevant. We recommend that clinicians facing similar situations maintain vigilance for signs of venous thromboembolism (e.g., deep or superficial vein thrombosis, pulmonary embolism), arterial thromboembolism (e.g., ischemia), and any degree of bleeding, even if minor. Given the significant mortality risk associated with both thrombocytopenia and withholding anticoagulation, the presence of any such findings should significantly influence decision-making. This case highlights a unique scenario in which clinical judgment must guide care in the absence of definitive guidelines. While severe, fluctuating thrombocytopenia would likely lead most providers to hold anticoagulation, the combination of several established prothrombotic risk factors complicates the situation. We hope this report encourages further documentation and discussion of similar cases to help develop specific guidelines, thereby facilitating improved patient-centered care.
